# Sigma-RF: prediction of the variability of spatial restraints in template-based modeling by random forest

**DOI:** 10.1186/s12859-015-0526-z

**Published:** 2015-03-21

**Authors:** Juyong Lee, Kiho Lee, InSuk Joung, Keehyoung Joo, Bernard R Brooks, Jooyoung Lee

**Affiliations:** 1 Laboratory of Computational Biology, National Heart, Lung, and Blood Institute, National Institutes of Health, 5635 Fishers Ln, Bethesda, 20852 USA; 2 Center for In Silico Protein Science, Korea Institute for Advanced Study, Seoul, Korea; 3 Center for Advanced Computation, Korea Institute for Advanced Study, Seoul, Korea; 4 School of Computational Sciences, Korea Institute for Advanced Study, Seoul, Korea

**Keywords:** Template-based modeling, Homology modeling, Random forest, Machine learning, Protein structure, Protein structure prediction, Protein sequence, Bioinformatics, Statistics

## Abstract

**Background:**

In template-based modeling when using a single template, inter-atomic distances of an unknown protein structure are assumed to be distributed by Gaussian probability density functions, whose center peaks are located at the distances between corresponding atoms in the template structure. The width of the Gaussian distribution, the variability of a spatial restraint, is closely related to the reliability of the restraint information extracted from a template, and it should be accurately estimated for successful template-based protein structure modeling.

**Results:**

To predict the variability of the spatial restraints in template-based modeling, we have devised a prediction model, Sigma-RF, by using the random forest (RF) algorithm. The benchmark results on 22 CASP9 targets show that the variability values from Sigma-RF are of higher correlations with the true distance deviation than those from Modeller. We assessed the effect of new sigma values by performing the single-domain homology modeling of 22 CASP9 targets and 24 CASP10 targets. For most of the targets tested, we could obtain more accurate 3D models from the identical alignments by using the Sigma-RF results than by using Modeller ones.

**Conclusions:**

We find that the average alignment quality of residues located between and at two aligned residues, quasi-local information, is the most contributing factor, by investigating the importance of input features used in the RF machine learning. This average alignment quality is shown to be more important than the previously identified quantity of a local information: the product of alignment qualities at two aligned residues.

**Electronic supplementary material:**

The online version of this article (doi:10.1186/s12859-015-0526-z) contains supplementary material, which is available to authorized users.

## Background

Due to the rapid increase of the size of the protein structure database, the template-based modeling has become a major tool for studying the structural aspect of proteins whose structures are not yet determined. Typical template-based modeling consists of three steps: 1) fold recognition, 2) sequence-template alignment and 3) chain building by optimizing spatial restraints. For the last decade, there have been significant improvements in the first two steps. A number of new methods have been proposed for improved fold recognition [1-6] and multiple sequence-template alignment [7-9] and these progresses have been validated in recent critical assessments of techniques for protein structure prediction experiments (CASPs) [10-14]. However, for the chain building step, the study of constructing accurate 3D models from a given alignment has not been as extensively explored as the other two steps [15], and the Modeller program [16,17] has been used as an efficient standard tool for many template-based modeling servers [2,18-21].

Generally, the chain building is carried out by optimizing a number of spatial restraints, which are extracted from a given sequence-template alignment. When a pair of residues in a target sequence are aligned with a corresponding pair in a template structure, the inter-atomic distance of the residue pair of the target sequence is assumed to take that of the template structure. The variability, denoted as *σ*, between two corresponding inter-atomic distances (*Δ* *d*=|*d*_*n*_−*d*_*t*_| where *d*_*n*_ is from the native structure and *d*_*t*_ from the template structure) is assumed to follow the Gaussian probability distribution function (PDF), which is defined as

$$p(\Delta d) = \frac{1}{\sigma \sqrt{2\pi}}\exp\left(-\frac{{\Delta d}^{2}}{2 \sigma^{2}}\right). $$

In Modeller [17] and Rosetta [22], the standard deviation of the PDF, *σ*, is estimated by fitting the Gaussian function against the histogram of data considering four features related to the quality of the alignment. For the chain building, the PDFs are converted into harmonic restraints by taking the negative logarithm and the model is constructed by minimizing the sum of the restraints. If a restraint is assumed to be more accurate than others, its corresponding *σ* value will be set to be relatively small and the restraint will be reinforced accordingly. Therefore, even with an identical alignment, the accuracy of a generated model would depend on the accuracy of *σ* values for the spatial restraints.

In this work, we have constructed a statistical prediction model, Sigma-RF, to predict the variability of the spatial restraint, 〈*Δ* *d*〉, by using the random forest (RF) method [23]. RF is an ensemble predictor, which consists of a number of decision trees and it makes a prediction from the ensemble average of outputs by individual trees. RF is one of the most accurate learning algorithms available and has been applied to many real-world problems [24-27]. It has advantages in dealing with large-size databases and many features [28]. The variability estimated by Sigma-RF, *σ*_RF_, has the following simple linear relationship with the standard deviation of Gaussian PDF used in Modeller, *σ*_*Modeller*_,

(1) $$ {\fontsize{8.8pt}{9.6pt}\selectfont{\begin{aligned} {}\langle \Delta d \rangle = \sigma_{\text{RF}} & = 2 \int_{0}^{\infty} \Delta d \: p(\Delta d) \: \mathrm{d}\Delta d \\ & = 2 \int_{0}^{\infty} \Delta d \frac{1}{\sigma_{Modeller}\sqrt{2\pi}}\exp\left(-\frac{{\Delta d}^{2}}{2 \sigma_{Modeller}^{2}}\right) \: \mathrm{d} \Delta d \\ & = \sqrt{\frac{2}{\pi}}\sigma_{Modeller}. \end{aligned}}}  $$

Therefore, the direct comparison of the correlation coefficients between the true variation of the distance restraint and the *σ* value either from Sigma-RF or Modeller is possible. When we benchmark the accuracy of *σ* prediction by Sigma-RF against that by Modeller, we find that the correlation between *σ* and *Δ* *d* by Sigma-RF outperforms that by Modeller when tested on 22 CASP9 targets, To identify the effect of the improvement of *σ* values, we performed template-based modeling of single-domain targets of recent CASP experiments: 22 from CASP9 and 24 from CASP10, by using the *σ* values from Sigma-RF (*σ*_*RF*_) and those from Modeller (*σ*_*Modeller*_) separately. The quality of model structures are compared in terms of the maximum TMscore [29] and lDDT-score [30] and the TMscore and lDDT-score of the minimum energy model. Single-domain template-based modeling targets of CASP9 and CASP10 were selected as benchmark targets. The importance of each input feature used in RF is estimated and its meaning and potential application to other related works are discussed.

## Methods

### Sequence-structure alignment preparation

To train Sigma-RF, a set of known sequence-structure alignments is necessary. To prepare a training set for Sigma-RF, a set of 1181 non-redundant protein sequences were selected from the PISCES server [31]. The criteria for filtering the non-redundant proteins are as follows: 1) sequence identity is less than 20%, 2) R-factor is less than 0.25, 3) structure is determined by X-ray and resolution is better than 1.6Å, 4) protein length ranges from 60 to 500 residues, and 5) there are no missing residues in the middle of a structure. The top-scoring template of each sequence was detected by an in-house fold recognition program, FoldFinder, which has been successfully used in previous CASP events [10,11,21,32]. FoldFinder is a profile-profile alignment tool using predicted secondary structure information of a target sequence by PSI-PRED [33], and predicted solvent accessibility by SANN [34]. In the fold database of FoldFinder, proteins released after CASP9 were eliminated for proper benchmarking.

### Feature generation

Following the Modeller procedure [16,17], for a given alignment between a target and its template, atom-pair distance information is extracted for all aligned residue pairs whose inter-atomic distance from the template structure is less than predetermined cutoff values. The pairs are grouped into four categories based on the atom-pair type: C *α*-C *α* (CACA), N-O (NO), Main chain-Side chain (MS), Side chain-Side chain (SS). The distance cutoff values of the four categories are 14.5, 10.0, 8.0 and 5.0Å, respectively. For a given pair of atoms, *i* and *j*, the variability of their spatial inter-atomic distance, the objective quantity for training, is defined as the difference between the distance from the native structure and the one from the template structure, $|d^{i,j}_{\textit {native}}-d^{i,j}_{\textit {template}}|$.

We considered 20 input features to train four random forest machines separately and they are described in Table 1. The first two features are related to the residue index difference between two aligned positions, *I* and *J*, in the target sequence. The third feature is the inter-atomic distance between atom *i* and atom *j* from two aligned positions in the template, *d*_*i*,*j*_. The fourth feature is the product of match scores of two aligned positions of target-template residues, *m*_*I*,*K*_ and *m*_*J*,*L*_, given by FoldFinder, which is equivalent to the local alignment quality in Rosetta [22]. The fifth feature is the average match score of two aligned positions and all aligned residues located between them. The four features, F6, F7, F10 and F11 are related to the number of gaps between two aligned positions in the target sequence (F6 and F7) and the template sequence (F10 and F11). The features, F8, F9, F12 and F13 are the reciprocals of the sequence distances from each aligned position to its closest gap. If a gap is placed next to an aligned residue, the value would be a unity, and it decreases monotonically as the distance from the gap increases.

**Table 1:** **20 input features used for Sigma-RF are listed along with their importance estimates**

**Index**	**Feature**	**Importance**
F1	|*I*−*J*|	7.51
F2	|*I*−*J*|/*N*_*res*,*target*_	2.91
F3	*d*_*template*_	9.43
F4	*m*_*I*,*K*_ *m*_*J*,*L*_	2.55
F5	$\sum _{\substack {I \leq i \leq J\\ K \leq j \leq L}} m_{i,j}\delta (i,j)/\sum _{\substack {I \leq i \leq J\\ K \leq j \leq L}}\delta (i,j)$	16.81
F6	$N_{\textit {gap}}^{IJ}$	1.91
F7	$N_{\textit {gap}}^{IJ}/|I-J|$	1.36
F8	1/|*I* ^′^−*I*|	0.12
F9	1/|*J* ^′^−*J*|	0.20
F10	$N_{\textit {gap}}^{KL}$	0.37
F11	$N_{\textit {gap}}^{KL}/|K-L|$	0.32
F12	1/|*K* ^′^−*K*|	0.23
F13	1/|*L* ^′^−*L*|	0.49
F14	$\sum _{s=H,E,C} p(s)\delta (s_{I},s_{K})$	0.16
F15	$\sum _{s=H,E,C} p(s)\delta (s_{J},s_{L})$	0.88
F16	$\sum _{acc=B,E} p(acc)\delta (acc_{I},acc_{K})$	0.53
F17	$\sum _{acc=B,E} p(acc)\delta (acc_{J},acc_{L})$	0.58
F18	*F*_4_ *F*_14_ *F*_15_ *F*_16_ *F*_17_	3.62
F19	$\frac {F_{18}}{1+F_{6}+F_{10}}$	3.02
F20	$\frac {F_{19}}{1+F_{8}+F_{9}+F_{12}+F_{13}}$	4.22

*I* and *J* (>*I*) indicate the residue indices in the target sequence, and *K* and *L* (>*K*) indicate those in the template sequence. When two residue pairs [(*I*, *K*) and (*J*, *L*)] are aligned, we extract the distance information of *d*_*template*_ between two atoms in the template. *N*_*res*,*target*_ is the chain length of the target sequence. *m*_*I*,*K*_ is the match score of the aligned pair (*I*, *K*). In F5, *δ*(*i*,*j*)=1, if residues *i*,*j* are aligned, otherwise *δ*(*i*,*j*)=0. $N^{I,J}_{\textit {gap}}$ is the number of gaps between *I* and *J* in the target sequence. *I* ^′^, *J* ^′^, *K* ^′^ and *L* ^′^ represent the residue indices of the closest gaps of *I, J, K* and *L*, respectively. *p(s)* represents the PSI-PRED scores of the secondary structure elements, helix (H), strand (E) and coil (C). *p(acc)* represents the SANN scores of the solvent accessibility states, buried (B) and exposed (E).

The next four features represent the consistency between the predicted secondary structure/solvent accessibility of the target and those of the template. In addition, we introduced three heuristic features, F18, F19 and F20, to consider correlations between features more explicitly. For example, F18 is defined as the product of match scores and consistency scores of two aligned positions since we observed that these features have positive correlations with the accuracy of the spatial restraint.

### Training random forest

The random forest algorithm is a machine learning algorithm using the ensemble of decision trees. Each tree is optimized by using a random subset of input features instead of deterministic optimization [35]. More detailed description of random forest can be found in the original reference [23]. We used the Breiman’s fortran 77 implementation of random forest, which can be downloaded from https://www.stat.berkeley.edu/~breiman/RandomForests/reg_examples/RFR.f.

From all sequence-template alignments in the training set, we could identify over 10 million pairs of aligned atoms whose inter-atomic distances were shorter than the corresponding cutoff value for each distance type. Among these, we selected 1 million cases randomly and used them to train a random forest machine. We trained 4 random forest machines considering 4 distance types separately: CACA, NO, MS, and SS. Each random forest consists of 200 decision trees. For each tree, 2/3 of the initial training set is randomly sampled with replacement to train the tree. The unused training set is called out of the bag (OOB) data and it is used to measure feature importance. At each split, 6 out of 20 features were randomly selected to find the best split that maximizes information gain [36]. The tree growth is stopped when 5 or less instances are included in the leaf node.

For prediction, a test case runs down all trees from the root to an end node based on the pre-determined splits. The output of each tree is defined as the average of *σ* values of instances included in the end node where the test case ends. The ensemble average of outputs from all 200 trees is considered as the final estimate of *σ* value.

The importance of each feature is measured by the increase of error in out of the bag (OOB) data after the value of the test feature among OOB data is permuted in a random fashion. When a tree is trained, the error of tree is estimated using the original OOB data. Next, the test feature is randomly permuted among the OOB data and the error of the tree is re-estimated by using the permuted data. The average difference between two error estimates over all trees in the forest is the raw importance score for the test feature.

### Homology modeling

Based on the linear relationship between the standard deviation (*σ*_*Modeller*_) of the Gaussian model of the distance restraint from Modeller [16] and our variability estimation (*σ*_*RF*_), the predicted variability can be utilized as the parameter of the harmonic spatial restraint to build a model structure, which is defined as

(2) $$ V(d_{ij})=\frac{1}{2}\left(\frac{d_{ij}-d^{'}_{ij}}{\sigma}\right)^{2},  $$

where *d*_*ij*_ and $d^{'}_{\textit {ij}}$ are distance between two atoms *i* and *j* in the model and in the template, respectively.

To test the influence of the accuracy of the restraint-distance variability on the quality of template-based modeling, we performed modeling of 22 CASP9 and 24 CASP10 targets by using *σ*_*RF*_ and *σ*_*Modeller*_. The best template of each target was detected by FoldFinder, and target-template alignments were obtained by MSA-CSA [7]. Protein structures released after the CASP9 and CASP10 experiment were excluded from the fold database of FoldFinder.

For a given alignment, a set of distance restraints was obtained by Modeller, and a new restraint file was generated by replacing *σ*_*Modeller*_ values of harmonic restraints with *σ*_*RF*_ values. Additionally, a restraint file generated by using the true *Δ* *d* values (*σ*_*native*_) is also prepared as the reference, which corresponds to the ideal prediction based on a given alignment.

For each target, 100 models were generated by executing restraint optimization with ModellerCSA [20] and original Modeller [16,37] using separate restraint files. The ModellerCSA package can be downloaded from http://lee.kias.re.kr/~protein/wiki/doku.php?id=modellercsa:download. It should be noted that, in this work, we excluded multiple binormal restraints of the Modeller energy function that affect the backbone and side-chain dihedral angles [37]. The quality of 3D models was evaluated by two measures, TM-score as the global quality measure [29] and lDDT-score–the local distance difference test score–as the local quality measure [30]. The maximum scores and the scores of the lowest energy conformations are compared.

## Results and discussion

### Prediction of structural variability

The correlation coefficients between the actual *Δ* *d*=|*d*_*n*_−*d*_*t*_| and the predicted variability values *σ* were calculated for 22 single-domain template-based modeling targets from CASP9. The results of four distance types, C *α*-C *α* (CACA), N-O (NO), main chain-side chain atoms (MS), and side chain-side chain atoms (SS), obtained by Sigma-RF and Modeller are shown in Table 2. Using Sigma-RF, clear and significant improvement of the average correlation coefficients for all four distance types is observed over Modeller results. The largest improvement is observed in MS restraints, which is increased from 0.187 to 0.458 and the improvement of CACA restraints which play the most important role in the chain building step is also considerable (increase from 0.226 to 0.355).

**Table 2:** **Correlation coefficients between predicted** ***σ*** **values and actual error, |** ***d***_***native***_ **−** ***d***_***template***_ **|, are shown**

**Target**	**Template**	**CACA**		**NO**		**MS**		**SS**	
		**Modeller**	**Sigma-RF**	**Modeller**	**Sigma-RF**	**Modeller**	**Sigma-RF**	**Modeller**	**Sigma-RF**
T0517	2qs7A	0.2912	**0.5622**	0.2492	**0.6013**	0.2016	**0.5217**	0.2614	**0.6158**
T0523	1ew0A	0.3518	**0.3923**	0.3382	**0.3621**	0.2505	**0.6017**	0.1397	**0.2932**
T0527	3f1pA	0.2131	**0.3402**	0.1347	**0.3309**	0.3624	**0.6456**	0.4031	**0.4859**
T0536	1ew0A	0.1969	**0.3138**	0.2261	**0.4194**	0.4438	**0.4754**	0.1690	**0.3075**
T0538	2kruA	0.1363	**0.2225**	0.1573	**0.2940**	-0.0174	**0.2370**	**0.1241**	-0.1121
T0539	1x4jA	0.2608	**0.5197**	0.2179	**0.5094**	0.2578	**0.3832**	-0.0061	**0.2398**
T0545	1wywA	0.1998	**0.3312**	0.1969	**0.3495**	0.2351	**0.5871**	**0.1435**	-0.1385
T0552	2q0zX	0.1053	**0.4061**	0.1197	**0.4386**	-0.0547	**0.5310**	0.0830	**0.5007**
T0557	3lmmA	0.2984	**0.4447**	0.3258	**0.4861**	-0.0896	**0.3577**	0.4558	**0.5855**
T0559	1qbjA	0.1473	**0.2589**	0.0865	**0.3176**	0.2635	**0.5315**	-0.2199	**0.1325**
T0560	2fokA	0.2076	**0.4392**	0.1677	**0.4554**	0.3255	**0.5524**	-0.0587	**0.1467**
T0566	1usuB	0.3187	**0.4536**	0.3524	**0.4407**	0.2816	**0.3639**	0.3723	**0.4313**
T0567	1ny5A	**0.2712**	0.1997	**0.2768**	0.2678	0.0558	**0.2472**	0.1784	**0.2378**
T0580	1iibA	0.0710	**0.2354**	0.1195	**0.3090**	-0.0505	**0.2871**	0.1281	**0.3200**
T0586	3by6A	0.1282	**0.3713**	0.0724	**0.3283**	-0.0420	**0.3020**	-0.1258	**-0.0214**
T0590	1l0qA	**0.1390**	0.1218	**0.1369**	0.0431	0.3497	**0.5478**	**0.3307**	-0.0593
T0594	1x53A	0.1894	**0.3364**	0.2257	**0.3768**	0.2010	**0.4539**	**0.2696**	0.1527
T0598	2osoA	0.2631	**0.3145**	0.3188	**0.3489**	0.2556	**0.4792**	0.3168	**0.3842**
T0610	1wdjA	0.2421	**0.2756**	0.2517	**0.3567**	0.2707	**0.5224**	0.3233	**0.4691**
T0615	1vj7A	0.3285	**0.4062**	0.3407	**0.5037**	0.1585	**0.4329**	0.2142	**0.3148**
T0622	3c1aA	0.3945	**0.5028**	0.4249	**0.4589**	0.2729	**0.5577**	0.2606	**0.4841**
Average		0.2264	0.3547	0.2257	0.3809	0.1872	0.4580	0.1792	0.2748

Better values are shown in bold face.

**Figure 1:**
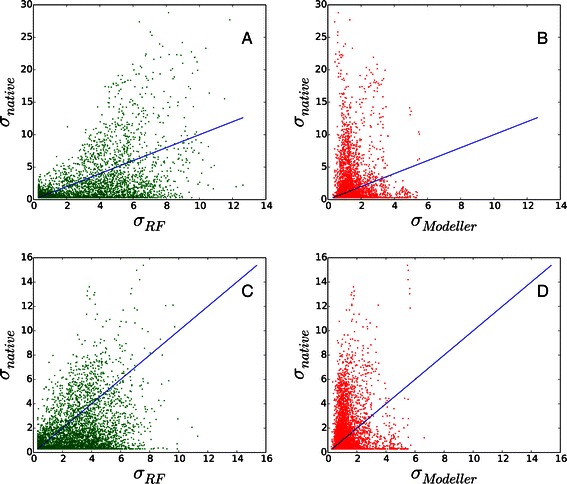
**Predicted distance variability values are shown against actual distance errors for T0552 and T0598.** The results of T0552 are shown in panel **A** and **B**, and those of T0598 are shown in panel **C** and **D**. The variability values by Sigma-RF, *σ*_*RF*_, (green) show better correlation with true distance deviations, *σ*_*native*_=|*d*_*native*_−*d*_*template*_|, than those by Modeller, *σ*_*Modeller*_, (red). The blue lines represent the linear correlation, *y*=*x*.

To illustrate details on the difference of results between Sigma-RF and Modeller, *σ* values of CACA restraints of T0552 and T0598 are shown in Figure 1. We observe that *σ*_*Modeller*_ (red) tend to have rather smaller values and they are more narrowly distributed than *σ*_*RF*_ (green). We note that many highly inaccurate spatial restraints, |*d*_*n*_−*d*_*t*_|>10.0Å, are assigned to have rather small *σ* values by Modeller, *σ*<2. These small *σ* values can significantly lower the accuracy of thus-generated 3D protein models since the corresponding harmonic restraints will cause large penalty scores for the native structure, which would prevent the sampling of more native-like conformations. On the other hand, Sigma-RF provides relatively larger *σ* values for highly inaccurate distance restraints than Modeller does. For T0552 and T0598, all highly inaccurate restraints are predicted with larger *σ* values by Sigma-RF. This will lower the penalty from inaccurate distance restraints and will potentially allow one to sample more native-like conformations, which are inaccessible with small *σ* values from Modeller.

One of the advantages of using RF is that we can estimate the importance of each input feature with little additional computational cost. We performed the importance test for 20 input features by using CACA restraints and the results are shown in Table 1. We find that the average match score of aligned residues located between and at two target positions, F5, is the most important factor for the variability prediction. Its importance score is significantly higher than the rest of the features. It is worth mentioning that this feature has not been considered previously either in Modeller [16] or Rosetta [22]. The second important feature is the spatial distance between two corresponding atoms in the template structure, which is considered both in Modeller [16] and Rosetta [22]. The third one is the residue index difference between two matched positions in the target sequence. These results indicate that the accuracy of distance information extracted from a template structure depends on the alignment quality of neighboring residues as well as that of two target positions. In addition, the distance information from the template is more reliable when physical and sequence distances between two target positions are relatively short.

In this work, we used three heuristic features, F18, F19 and F20, to take into account the relationship between features more explicitly. F18 is the product of match scores of two target positions successively multiplied by consistency scores considering the predicted secondary structure and solvent accessibility between the target and the actual value from a template. All features used to generate F18 are expected to be positively correlated with the local similarities at target positions. Therefore, as the characteristics of target positions are consistent between predicted values and actual values, this feature’s relevance will increase. F19 is defined as the division of F18 by (1+F6+F10), where F6 and F10 is the number of gaps between two target positions of the target and the template, respectively. The number of gaps in the alignment is expected to correlate negatively with the accuracy of the alignment, and therefore the smaller value of F19 would indicate that the local alignment between two target positions is less reliable. Similarly, F20 is defined as the division of F19 by (1+F8+F9+F12+F13).

For F8, F9, F12 and F13, the reciprocal to the closest gap is defined as zero if there are no neighboring gaps, i.e., these features become zero. The distance from an aligned position to its closest gap is also closely related to the accuracy of alignment. This was identified as the most [16] or the second most [22] important feature among four features in previous studies. As the closest gap is located further from two target positions, F20 increases. The relatively high importance values of these features (F18, F19, F20) than the individual features used to generate them (see Table 1) demonstrate that devising an intuitive heuristic feature can be useful in reducing the complexity and computational time when dealing with a large number of input features. The importance of individual similarity and gap-related features appears to be relatively low, since the essence of equivalent information is already considered.

Next, we tested the performance of the machine trained by using only the 10 most importance features to validate the importance estimates. The correlation coefficient between the true and predicted *σ* values of the CACA restraints are shown in Table 3. Only slight decrease of the average correlation coefficient was observed by using the top 10 features from 0.355 to 0.339. The excluded low-importance features are related to the distance from a gap and the secondary structure/solvent accessible area information. This indicates that the importance estimate obtained by the random forest is quite reliable and the information contained in the excluded features can be mostly captured by a smaller number of heuristic variables, F18, F19 and F20.

**Table 3:** **Correlation coefficients between predicted** ***σ*** **values by Sigma-RF and the actual errors for CACA distances of 22 CASP9 targets are shown**

**Target ID**	**With 20 features**	**With top 10 features**
T0517	0.5622	0.5774
T0523	0.3923	0.3041
T0527	0.3402	0.3355
T0536	0.3138	0.3438
T0538	0.2225	0.2998
T0539	0.5197	0.5093
T0545	0.3312	0.2289
T0552	0.4061	0.4277
T0557	0.4447	0.3720
T0559	0.2589	0.2237
T0560	0.4392	0.4080
T0566	0.4536	0.3619
T0567	0.1997	0.1960
T0580	0.2354	0.2948
T0586	0.3713	0.4038
T0590	0.1218	0.0670
T0594	0.3364	0.3330
T0598	0.3145	0.2489
T0602	0.5608	0.4723
T0610	0.2756	0.2825
T0615	0.4062	0.4177
T0622	0.5028	0.4853
Average	0.3640	0.3452

Results using the full 20 features as well as using top 10 features are shown. On average, by using only half of the features, 95% of the prediction level is achieved.

### Application to homology modeling

**Figure 2:**
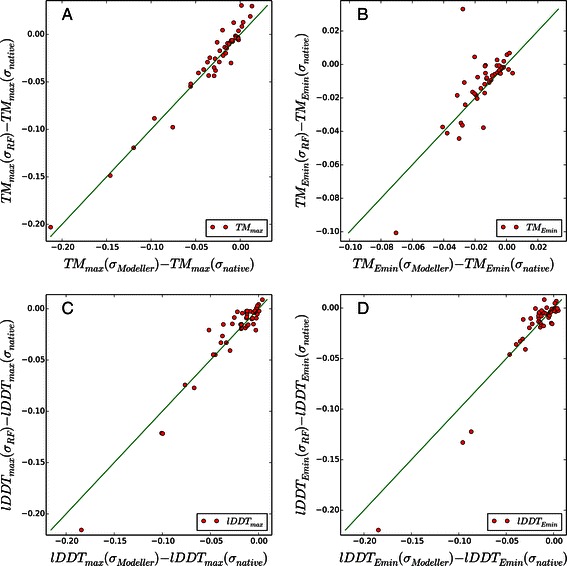
**A comparison of TM-scores and lDDT-scores of 3D models generated by ModellerCSA using** ***σ***_***RF***_ **and** ***σ***_***Modeller***_ **from those using** ***σ***_***native***_ **.** The TM-score results are shown in panel **A** and **B**, and the lDDT-score results are shown in panel **C** and **D**. For all plots, X-axes represent the quality measure differences between models obtained by *σ*_*Modeller*_ and *σ*_*native*_. Y-axes represent the differences between models obtained by *σ*_*RF*_ and *σ*_*native*_. The green lines represent the *y*=*x* line, which corresponds to the identical model quality. The number of dots over the green line corresponds to the targets that are improved by using *σ*_*RF*_.

The average model quality measures of homology modeling results of 46 benchmark targets obtained by ModellerCSA using *σ*_*RF*_, *σ*_*Modeller*_ and *σ*_*native*_ are summarized in Table 4. About 70% of benchmark targets are improved in terms of TM-score measures (the upper panels of Figure 2 and Additional file 1). The average *T* *M*_*max*_,*T* *M*_*Emin*_ and *T* *M*_*avg*_ values obtained with *σ*_*RF*_ are consistently higher than those with *σ*_*Modeller*_.

**Table 4:** **Average model quality measures of homology modeling results of 46 benchmark targets obtained by ModellerCSA using** ***σ***_***RF***_ **,** ***σ***_***Modeller***_ **, and** ***σ***_***native***_ **are shown**

	***T*** ***M***_***max***_	***T*** ***M***_***Emin***_	***T*** ***M***_***avg***_	***l*** ***D*** ***D*** ***T***_***max***_	***l*** ***D*** ***D*** ***T***_***Emin***_	***l*** ***D*** ***D*** ***T***_***avg***_
*σ*_*native*_	0.756	0.734	0.710	0.661	0.650	0.648
*σ*_*RF*_	0.730	0.722	0.707	0.636	0.630	0.626
*σ*_*Modeller*_	0.727	0.719	0.691	0.635	0.630	0.624
No. of improved targets	32/46	33/46	34/46	29/46	29/46	30/46

In terms of lDDT-score measures, 63% of targets improved although the average values are almost identical (the lower panels of Figure 2 and Additional file 2). The lesser improvement in lDDT-scores may originate from less accurate *σ* predictions of SS atom-pairs than those of main-chain related atom-pairs (Table 2). These results show that using *σ*_*RF*_ for the chain-building step during protein structure prediction can consistently lead to better models than using *σ*_*Modeller*_ for a given sequence-template alignment with little additional computational cost.

**Figure 3:**
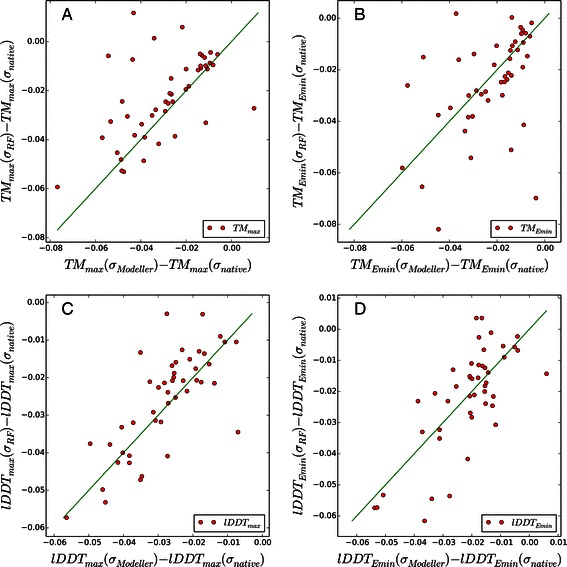
**A comparison of TM-scores and lDDT-scores of 3D models generated by Modeller using** ***σ***_***RF***_ **and** ***σ***_***Modeller***_ **from those using** ***σ***_***native***_ **.** The TM-score results are shown in panel **A** and **B**, and the lDDT-score results are shown in panel **C** and **D**. For all plots, X-axes represent the quality measure differences between models obtained by *σ*_*Modeller*_ and *σ*_*native*_. Y-axes represent the differences between models obtained by *σ*_*RF*_ and *σ*_*native*_. The green lines represent the *y*=*x* line, which corresponds to the identical model quality. The number of dots over the green line corresponds to the targets that are improved by using *σ*_*RF*_.

We also performed the homology modeling of benchmark targets using the original Modeller package to identify whether predicting better *σ* value is useful without using ModellerCSA (Table 5 and Additional file 3 and 4). The results show that using *σ*_*RF*_ with Modeller significantly improves the quality of the best model. The *T* *M*_*max*_ values of 36 targets improved (Figure 3A). However, unlike the results of ModellerCSA, other measures, *T* *M*_*Emin*_, *T* *M*_*avg*_, *l* *D* *D* *T*_*Emin*_ and *l* *D* *D* *T*_*avg*_ values are showing no improvement (the middle and right panels of Figure 3). This difference may be attributed to the lack of extensive conformational sampling. ModellerCSA performs much more extensive conformational sampling than Modeller and always finds lower energy conformations. Thus the minimum energy conformations obtained by Modeller are likely to be remote from the true energy minimum, which makes *T* *M*_*Emin*_ results less meaningful.

**Table 5:** **Average model quality measures of homology modeling results of 46 benchmark targets obtained by original Modeller using** ***σ***_***RF***_ **,** ***σ***_***Modeller***_ **, and** ***σ***_***native***_ **are shown**

	***T*** ***M***_***max***_	***T*** ***M***_***Emin***_	***T*** ***M***_***avg***_	***l*** ***D*** ***D*** ***T***_***max***_	***l*** ***D*** ***D*** ***T***_***Emin***_	***l*** ***D*** ***D*** ***T***_***avg***_
*σ*_*native*_	0.764	0.744	0.743	0.635	0.617	0.616
*σ*_*RF*_	0.741	0.719	0.719	0.609	0.595	0.593
*σ*_*Modeller*_	0.735	0.721	0.719	0.607	0.595	0.592
No. of improved targets	36/46	21/46	22/46	27/46	22/46	29/46

A comparison of ModellerCSA and Modeller results shows that the Modeller results are more accurate in terms of the TM-scores. The higher TM-scores of Modeller results may be due to the difference in energy functions. ModellerCSA used a modified Modeller energy function without multiple binormal restraints that consider backbone and side-chain dihedral angle preferences. However, the ModellerCSA results are showing higher lDDT-scores, which correspond to more accurate side-chain conformations. This significant improvement in side-chain conformations is consistent with what was observed in previous ModellerCSA study [20]. Model quality improvement by sampling lower Modeller energy was more prominent in side-chains accuracy than backbone accuracy.

**Figure 4:**
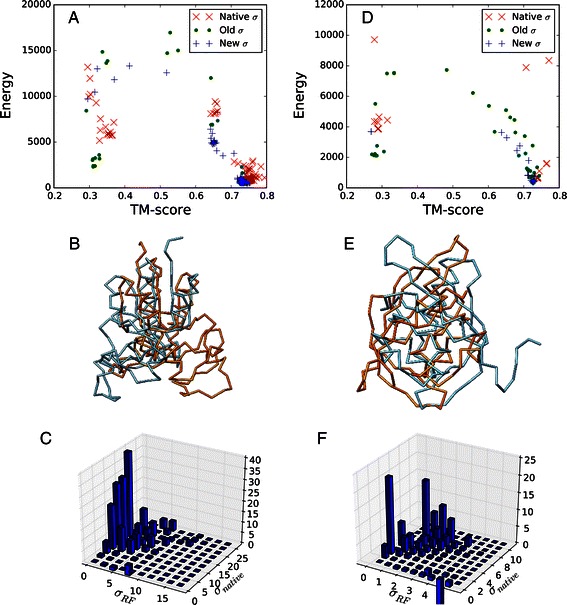
**A comparison of template-based modeling results of T0517 and T0523 by the** ***σ***_***RF***_ **and** ***σ***_***native***_ **values.** The energy landscapes of template-based modeling results of **(A)** T0517 and **(D)** T0523 by *σ*_*RF*_, *σ*_*Modeller*_ and *σ*_*native*_. The representative structures of low and high TM-score results are superposed: **(B)** T0517 and **(E)** T0523. The average restraint energy differences, *E*_*RF*_−*E*_*native*_, of the mirror-image structures of **(C)** T0517 and **(F)** T0523 evaluated by *σ*_*RF*_ and *σ*_*native*_ are shown as 3D histogram plots. Positive z-axis values indicate that corresponding distance restraints are favored by *σ*_*native*_ and disfavored by *σ*_*RF*_.

It should be noted that, for some targets, the average TM-scores of *σ*_*RF*_ results are even higher than those of *σ*_*native*_ results. To identify the reason for this unintuitive result, we examined the energy landscapes of two targets, T0517 and T0523 (see Figure 4). From the energy landscapes (Figure 4A and 4D), it is clear that final 100 conformations are clustered into two groups for all three cases of *σ*. The majority of conformations are located near TM-score=0.75 with lower energies while some conformations are located near TM-score=0.3 with higher energies. The superposition of structures from the two regions shows that the lower TM-score structures correspond to mirror images of more native-like structures (see Figure 4B and 4E). The occurrence of mirror-images has been observed in many other modeling approaches based on the optimization of distance restraints [38-41].

The energy landscapes show that a smaller number of conformations are found in the low TM-score region by using *σ*_*RF*_, which suggests that the distance restraints by *σ*_*RF*_ energetically disfavor the formation of mirror-images. To validate this assumption, the restraint energy differences between *σ*_*RF*_ and *σ*_*native*_, $\Delta E_{\textit {ij}} = E_{\textit {ij}}^{RF} - E_{\textit {ij}}^{native}$ where $E_{\textit {ij}}^{RF}$ and $E_{\textit {ij}}^{native}$ are respectively distance restraint energies between atom *i* and *j* by *σ*_*RF*_ and *σ*_*native*_, are calculated for the mirror images of T0517 and T0523 (see Figure 4C and 4F). The plots demonstrate that, for the mirror images of T0517 and T0523, distance restraints with large *σ*_*native*_ are penalized more by *σ*_*RF*_ than by *σ*_*native*_. In the case of T0517, there are a number of restraints whose *σ*_*native*_ values are over 10 Å due to erroneous target-template alignment. With larger *σ*_*native*_ values than *σ*_*RF*_ values, the residues related to these restraints experience less unfavorable restraint energies and are modeled almost freely, which may allow it to adopt a mirror-image structure without causing much penalty. Similarly, in the mirror-image of T0523, the distance restraints with *σ*_*native*_>4Å become energetically much more unfavorable by *σ*_*RF*_. This observation is consistent with a previous NMR study, which reported that the likelihood of obtaining an inverted structure is higher when the number of restraints is insufficient [38]. This explains why using large *σ*_*native*_ values for poorly aligned regions tends to result in more mirror-image structures.

In summary, for a given sequence-template alignment, chain building using *σ*_*RF*_ leads to more accurate protein modeling than that using *σ*_*Modeller*_ in terms of local atomic details as well as the global structure.

### Advantages of random forest

In this work, we have used the random forest learning algorithm to predict the variability of the spatial restraint in the template-based modeling. The random forest method has a number of advantageous features: 1) it is one of the most accurate learning algorithms available, 2) it can handle large datasets efficiently, 3) it can handle a large number of input features without modification or deletion and 4) it provides an estimate of importance for each input feature [23]. In previous sigma prediction studies [16,22], histogram-based approaches were used, where a database was constructed by dividing and storing the learning instances into the bins of input feature space and the width of the Gaussian PDF of sigma was fitted on the histogram of instances.

One shortcoming of the histogram approach is that the number of input features and the number of bins are limited by the size of the database. If there are 5 input features each of which is divided into 10 bins, a total of 100,000 bins should be considered, which would require at least 10 million data points to obtain a reasonable estimate of the quantity of interests. The size of database can increase even further by including an additional feature. In addition, a considerably large size of the database does not always guarantee that all bins are properly filled. Therefore, to obtain an accurate estimation of *σ* values using the histogram-based approach, one should be careful in selecting only a small number of relevant input features, the identities of which are generally unknown in advance. By using the random forest method, however, we were able to use as many as 20 input features readily.

The random forest method can measure the importance of each feature during the training with a fraction of additional computational cost. The importance estimation of an input feature can uncover hidden relationships between local properties of protein attributes. We found that the average match score of all aligned residue pairs located between and at two target positions is the most relevant information to predict the accuracy of the distance restraint extracted from the template. This suggests that the alignment quality of two target positions depends on their neighboring residues as well as the aligned pair themselves. This feature has not been considered in existing homology modeling studies [16,22].

Therefore, incorporating the equivalent information may help to improve the accuracy of Modeller [17] and/or Rosetta [22]. In addition, the minimal increase of the prediction accuracy of the machine trained with all twenty features over the one using only top 10 features suggests that the importance estimation of the current random forest implementation is quite reliable, and it can serve as a useful tool to analyze and simplify problems in related bioinformatics.

### Beyond this work

Additional improvement in the model quality can be achieved by using a multiple sequence alignment. In this work, the single template alignment of each target was used to measure the sole effect of new *σ* values on the 3D chain building. However, in general, it is well known that the multiple alignment can help to generate more accurate protein 3D models. By using the multiple alignment and Sigma-RF, the modeling quality of such residues, which are aligned in terms of multiple templates, are likely to improve the model quality even further if accurate *σ* values are assigned to competing distance restraints originating from separate templates. Obviously, accurate assignment of *σ* values will allow thus-generate model to adopt the more accurate part selectively out of multiple template structures.

## Conclusion

In this work, we have trained a statistical model, Sigma-RF, to predict the intrinsic variability of the distance restraint between a residue pair using the random forest algorithm. Benchmark results show that Sigma-RF predictions are more highly correlated with the true variability than Modeller results. The homology modeling of 46 CASP9 and CASP10 targets shows that the utilization of the variability predicted by Sigma-RF consistently leads to more accurate three-dimensional protein models than using Modeller predictions with the identical alignment. The importance test of input features shows that the average alignment quality of residues located between and at two aligned residues, quasi-local information, is the most important feature in determining the variability of the distance restraint. This average alignment quality is shown to be more important than the previously identified quantity of local information: the product of alignment qualities at two aligned residues.

## Additional files

Additional file 1
**The TM-score measures of ModellerCSA results using** ***σ***_***native***_ **,** ***σ***_***RF***_ **, and** ***σ***_***Modeller***_ **are shown.** An excel file contains the complete list of TM-score measures, *T* *M*_*max*_,*T* *M*_*Emin*_ and *T* *M*_*avg*_, of ModellerCSA results of 46 CASP9 and CASP10 benchmark targets.

Additional file 2
**The lDDT-score measures of ModellerCSA results using** ***σ***_***native***_ **,** ***σ***_***RF***_ **, and** ***σ***_***Modeller***_ **are shown.** An excel file contains the complete list of TM-score measures, *l* *D* *D* *T*_*max*_,*l* *D* *D* *T*_*Emin*_ and *l* *D* *D* *T*_*avg*_, of ModellerCSA results of 46 CASP9 and CASP10 benchmark targets.

Additional file 3
**The TM-score measures of Modeller results using** ***σ***_***native***_ **,** ***σ***_***RF***_ **, and** ***σ***_***Modeller***_ **are shown.** An excel file contains the complete list of TM-score measures, *T* *M*_*max*_, *T* *M*_*Emin*_ and *T* *M*_*avg*_, of Modeller results of 46 CASP9 and CASP10 benchmark targets.

Additional file 4
**The lDDT-score measures of Modeller results using** ***σ***_***native***_ **,** ***σ***_***RF***_ **, and** ***σ***_***Modeller***_ **are shown.** An excel file contains the complete list of TM-score measures, *l* *D* *D* *T*_*max*_,*l* *D* *D* *T*_*Emin*_ and *l* *D* *D* *T*_*avg*_, of Modeller results of 46 CASP9 and CASP10 benchmark targets.
